# Reconstructing IDF curves from daily rainfall records in data-scarce regions: A statistical method based on temporal disaggregation and gumbel modeling

**DOI:** 10.1371/journal.pone.0351841

**Published:** 2026-07-09

**Authors:** Jay Molino, Humberto Martí-Fis, Yakelin Rodriguez-López

**Affiliations:** 1 Universidad Especializada de las Américas (UDELAS), Faculty of Biosciences and Public Health, Centro I+D+i de Biotecnología, Energías Verdes y Cambio Climático, Panama, Panama; 2 Centro de Investigaciones Hidráulicas, Universidad Tecnológica de La Habana José Antonio Echeverría, CUJAE, Havana, Cuba; CEEW: Council on Energy Environment and Water, INDIA

## Abstract

Urban regions in the tropics often face challenges in hydrological design due to the lack of high-resolution rainfall data. This study presents a method for generating intensity, duration, frequency (IDF) curves using only daily rainfall records, applied to the case of Habana del Este, Cuba. Daily maximum rainfall data from eight pluviometric stations (2010–2024) were combined with subdaily observations from a reference pluviograph. Two strategies were used: direct integration for compatible stations and temporal disaggregation for incompatible ones. Rainfall intensities for return periods between 2 and 1000 years were estimated using Gumbel frequency analysis and fitted to the Sherman model through nonlinear regression. The resulting IDF curves were unified into a single regional model using a weighted average of rainfall intensities from the compatible and disaggregated station groups, followed by final Sherman model fitting. The main contribution of this study is an integrated reconstruction framework that links station compatibility screening, selective use of observed subdaily rainfall structure, disaggregation of non-compatible daily records, and weighted regional unification within a single reproducible workflow. The final curves showed excellent agreement across durations and return levels (R^2^ > 0.998), with strong internal consistency between estimation methods. Validation using linear and log log plots confirmed the robustness of the approach. This method provides a practical and statistically sound solution for IDF curve development in data scarce tropical regions, offering direct support for infrastructure planning, hydraulic design, and climate resilience where subdaily rainfall observations are limited.

## Introduction

Extreme rainfall events present a growing challenge for hydrological infrastructure, particularly in urban areas and regions exposed to climate variability [1–5]. These events, often short in duration yet high in intensity, can overwhelm drainage systems and lead to flash flooding, infrastructure failure, and significant socioeconomic losses. Accurately estimating short-duration rainfall intensities is therefore essential for the design of stormwater networks, flood protection systems, urban roadways, and sewer systems [2,4,6]. The ability to anticipate rare but intense storms is fundamental for planning resilient cities under both current and future climate conditions.

A standard tool for hydrological design is the intensity–duration–frequency (IDF) curve, which relates the statistical probability of a rainfall event exceeding a given intensity over a defined duration and return period [3,7,8]. These curves allow engineers to determine design storms for hydraulic structures based on local climatology and risk tolerance. However, constructing IDF curves traditionally requires long-term high-resolution rainfall data (e.g., 5–60-minute intervals), which are unavailable in many developing or data-scarce regions [1,4–6,9]. In such cases, planners face the dilemma of either importing intensity-duration values from other areas or relying on approximations that may not reflect local hydrometeorological conditions.

To overcome this data gap, researchers have explored statistical and empirical methods to estimate IDF curves from daily precipitation records, which are more commonly available. These approaches include empirical temporal disaggregation, scaling laws, and extreme value modeling tailored to longer-duration datasets [6,10–13]. Daily rainfall series, often covering multiple decades, serve as a feasible surrogate for high-frequency data, particularly in parts of Latin America, Africa, and Asia where dense pluviograph networks are absent or have only recently been established [4,7,9]. This makes them attractive inputs for simplified methods of IDF curve generation that retain statistical rigor while minimizing data requirements.

Among the various approaches, temporal disaggregation techniques are widely adopted due to their simplicity, data efficiency, and adaptability to local or regional contexts. These methods rely on fixed or probabilistically derived ratios to redistribute daily rainfall totals into synthetic sub-daily intervals (e.g., 5, 10, 30, 60, or 120 minutes) [6,10,14–16]. The disaggregation ratios are typically based on climatological norms, regional studies, or statistical analysis of higher-resolution datasets where available. By generating internally consistent sub-daily rainfall sequences, such methods enable frequency analysis of rare events without the need for direct observation at finer temporal scales.

Numerous studies have implemented such techniques to generate synthetic sub-daily rainfall time series and derive IDF relationships across data-scarce regions. Applications have been documented in Brazil, Colombia, Iraq, South Africa, Uganda, and other countries where ground-based sub-daily measurements are limited [6–9,12,17]. Validation efforts generally report acceptable levels of accuracy, with median error margins often within 10–20% compared to observed sub-daily extremes. However, some underestimation of peak intensities at very short durations remains a known limitation, underscoring the importance of careful calibration and regional adaptation.

Following disaggregation, the derived sub-daily maxima are subjected to frequency analysis using statistical models. The most commonly applied distributions include the Gumbel (EV1) and the Generalized Extreme Value (GEV) families [1,3,12,14,15,18]. These models are fitted to annual maximum series (AMS) or partial duration series (PDS) depending on the dataset and design requirements. Selection of the best-fit distribution is usually guided by statistical tests such as the Kolmogorov–Smirnov, Anderson–Darling, or Akaike Information Criterion, with attention paid to quantile accuracy and tail behavior [13,14].

In recent years, new data sources and computational tools have expanded the possibilities for IDF curve estimation in ungauged or poorly gauged regions. Satellite precipitation products (e.g., TRMM, GPM, IMERG), reanalysis datasets (e.g., ERA5, ERA5-Land), and radar-based QPE offer quasi-global coverage and temporal resolution suitable for rainfall frequency analysis [2,10,16,19]. When combined with local gauge correction or bias adjustment procedures, these datasets have proven valuable for extending or supplementing observed records, especially in countries with fragmented hydrometeorological networks.

Complementary to these efforts, modern approaches increasingly leverage machine learning and Bayesian frameworks to model rainfall extremes under non-stationary climate conditions. Techniques such as artificial neural networks, random forests, and hierarchical Bayesian modeling have been applied to disaggregate rainfall, fit probability distributions, and simulate future IDF curves under different climate scenarios [13,20,21]. These innovations seek to improve the robustness and transferability of IDF estimation methods while acknowledging the effects of climate change on rainfall behavior.

Accordingly, this study proposes and validates a method to reconstruct IDF curves from daily rainfall data. The approach combines compatibility screening, temporal disaggregation, Gumbel frequency analysis, Sherman model fitting, and weighted regional unification. It was applied to a 15-year dataset of daily maximum rainfall records from Habana del Este, Cuba, covering the period 2010–2024, and is intended for broader use in other data-scarce regions where sub-daily observations are lacking. The proposed methodology emphasizes accessibility, simplicity, and replicability in contexts where resource constraints make traditional pluviograph-based analyses impractical.

## Theory

The analytical framework employed in this study comprises three core components: frequency analysis using the Gumbel distribution, intensity–duration–frequency (IDF) curve fitting via the Sherman model, and exponential temporal disaggregation of daily rainfall. These tools form the mathematical basis for estimating sub-daily rainfall intensities from daily records in regions lacking high-resolution observations.

### Gumbel distribution for frequency analysis

To estimate rainfall depths associated with different return periods, this study employs the Gumbel distribution, commonly used to model the behavior of annual maximum rainfall events. Its cumulative distribution function (CDF) is given by:


$$\begin{array}{c} F(x)=e^{{-e}^{-\left[\frac{x-\mu }{\beta }\right]}} \end{array}$$

where x is the annual maximum rainfall, and μ and β are the location and scale parameters, respectively. From this distribution, the rainfall depth x_T_ corresponding to a return period T is computed as:


$$\begin{array}{c} x_{T}=\mu -\beta \cdot ln\left[-ln\left(\text{1}-\frac{\text{1}}{T}\right)\right] \end{array}$$

These estimates constitute the foundation for IDF curve development. Since daily data were used, the Gumbel parameters were derived via the method of moments using the sample mean and standard deviation:


$$\begin{array}{c} \mu =\tilde{x}-\gamma \cdot \beta ,\;\beta =\frac{s\cdot \sqrt{\text{6}}}{\pi } \end{array}$$

with γ ≈ 0.5772 (Euler–Mascheroni constant). The return level estimates x_T_ serve as input for subsequent curve fitting and disaggregation.

### Sherman equation for IDF curve fitting

To express rainfall intensity as a function of duration and return period, the study utilizes the Sherman model:


$$\begin{array}{c} i(t)=\frac{a}{(t+b{)}^{c}} \end{array}$$

This empirical equation allows transforming rainfall depths into intensities over various storm durations t, with a, b, and c serving as calibration parameters for each return period. It provides a flexible, continuous function well-suited to hydrological design applications.

The model was fitted using nonlinear regression, and where appropriate, logarithmic transformation:


$$\begin{array}{c} \text{ln}(i)=\text{ln}(a)-c\cdot \text{ln}(t+b) \end{array}$$

This curve-fitting process bridges the statistical estimation of rainfall magnitudes and the engineering requirement for duration-based intensity inputs.

### Temporal disaggregation of daily rainfall

To reconstruct sub-daily intensities from daily records, the study applies an exponential disaggregation model that estimates the cumulative proportion of rainfall accumulated over time:


$$\begin{array}{c} P(t)=\text{1}-e^{-kt} \end{array}$$

Here, k controls the rate at which the daily rainfall depth is redistributed into shorter durations. In this study, k = 0.45 was adopted as an empirical disaggregation exponent suitable for humid tropical rainfall regimes, particularly those influenced by convective and convective stratiform storm structures. This value was not treated as a universal constant or as the result of a full local pluviograph calibration. Rather, it was selected based on hydrological guidance and previous regional applications for tropical rainfall disaggregation, and its use was subsequently evaluated by assessing the internal consistency of the resulting IDF curves. This function allows partitioning daily rainfall totals h24 into shorter durations:

$h_{t}=P(t)\cdot h_{\text{2}\text{4}},\;i_{t}=\frac{h_{t}}{t}$

This approach ensures that the integrity of the daily volume is preserved while enabling frequency analysis at durations relevant to infrastructure design (e.g., 5–120 minutes). The simplicity of the model facilitates its use in regions with limited data and computational resources.

### Weighted parameter aggregation (regionalization step)

To enhance spatial representativeness, the study computes regional IDF parameters by aggregating station-specific Sherman coefficients using weighted averages:


$$\begin{array}{c} \overset{―}{a}=\sum_{i=\text{1}}^{n}w_{i}a_{i},\;\overset{―}{b}=\sum_{i=\text{1}}^{n}w_{i}b_{i},\;\overset{―}{c}=\sum_{i=\text{1}}^{n}w_{i}c_{i} \end{array}$$

Weights w_i_ reflect the statistical reliability of each station and ensure that final curves are not dominated by outliers. This aggregation forms the basis for a unified, regional IDF curve, supporting the study’s goal of providing scalable methodologies for data-scarce hydrological contexts.

## Materials and methods

This study was conducted in Habana del Este, a coastal municipality in Cuba characterized by a tropical humid climate with pronounced seasonality and exposure to extreme weather events. The area’s vulnerability to intense rainfall and the limited availability of high-resolution rainfall data motivated the development of a methodology capable of generating reliable IDF curves using only daily precipitation records.

The methodological framework consisted of three integrated phases: (1) data collection, validation, and reconstruction; (2) frequency analysis and compatibility assessment; and (3) IDF curve construction and unification. All stages were grounded in established hydrological practices and tailored to the data conditions of the study area.

### Phase 1: Data collection, quality control, and series reconstruction

Daily maximum rainfall data for the period 2010–2024 were obtained from the Sistema de Gestión Integral del Agua (SGIA), Cuba’s official hydrometeorological platform. No extension of the dataset prior to 2010 was performed. Initially, ten pluviometric stations located within or near Habana del Este were considered. A preliminary quality check eliminated two stations with insufficient temporal coverage or unreliable records. The remaining eight stations were subjected to homogeneity analysis using the coefficient of variation (CV), comparing the relative dispersion of each series against the group mean. Seven stations met the ± 15% homogeneity criterion; the eighth station, which initially showed anomalous dispersion due to incomplete annual maximum values within the 2010–2024 period, was reconstructed using the mean ratio method and subsequently reevaluated for homogeneity.

Reconstruction of missing years in this station was performed using the mean ratio method. This involved calculating the average annual maximum rainfall for the incomplete station during years with available data and comparing it to the average of a reference group of homogeneous stations during the same period. The ratio of these means served as a correction factor to estimate missing values, year by year, based on the observed data in the reference group. The reconstructed series was subsequently reevaluated for homogeneity and included in the subsequent analyses.

### Phase 2: Frequency analysis and compatibility testing

All annual maximum series, now complete and homogeneous, were fitted to the Gumbel distribution using the method of moments. The location and scale parameters were derived from the sample mean and standard deviation, enabling the estimation of rainfall magnitudes associated with return periods of 2, 5, 10, 25, 50, 100, and 1000 years.

To determine the integration strategy for each station, a compatibility assessment was conducted by comparing the estimated 24-hour precipitation values of each station with those of the reference pluviograph station. The latter provided a previously established IDF curve fitted from sub-daily observations. The relative percentage difference between the 24-hour values across return periods served as the compatibility metric. Stations whose deviations remained below 15% were classified as hydrologically consistent with the pluviograph and were directly integrated into the composite curve construction.

### Phase 3: IDF curve construction, disaggregation, and unification

For compatible stations, the estimated daily rainfall intensities were averaged for each return period and used to update the 1440-minute (24-hour) point in the reference IDF curve. The rest of the sub-daily values from the pluviograph were retained. This combined dataset was used to recalibrate the Sherman equation for each return period using nonlinear regression, minimizing the root mean square error (RMSE) and ensuring smooth decay in intensity with increasing duration.

Stations found incompatible with the pluviograph were processed separately using a temporal disaggregation procedure. The adopted model, based on an exponential decay function calibrated with k = 0.45, redistributed each 24-hour precipitation total into shorter durations, preserving volume and reflecting typical storm structures in tropical climates. Disaggregated values were computed for durations ranging from 10 to 1440 minutes and used to construct synthetic IDF tables. These were also fitted to the Sherman equation, ensuring consistency across durations and return periods.

Finally, both sets of curves, those derived from compatible stations and those generated through disaggregation, were integrated into a unified IDF dataset. For each duration and return period, rainfall intensities were averaged using a weighted scheme based on the number of contributing stations in each group. This regionalization step enhanced the spatial representativeness of the final curve while preserving the statistical integrity of its components. The resulting composite IDF relationship was recalibrated through Sherman model fitting, yielding a single continuous and validated function for engineering use in Habana del Este and potentially applicable to similar data-scarce urban settings.

## Results

The construction of IDF curves for Habana del Este resulted in a unified model that integrates sub-daily pluviograph records with daily data from eight pluviometric stations (2010–2024). Quality control procedures confirmed that seven stations met the ± 15% homogeneity criterion relative to the mean coefficient of variation, while one incomplete station was reconstructed using the mean ratio method and subsequently reevaluated. A Gumbel distribution provided the best fit to the annual maxima, allowing 24-hour quantile estimation for return periods of 2–1000 years. Three stations showed deviations below 15% with respect to the CH-345 reference and were incorporated through composite averaging. The remaining stations were integrated via exponential disaggregation (k = 0.45). The combination of these datasets yielded a consistent regional IDF curve, reflecting both spatial and temporal rainfall variability in the study area.

Table 1 (shown below), presents the unified rainfall intensities (mm/h) for durations ranging from 10 to 1440 minutes across seven return periods:

**Table 1:** Unified rainfall intensities for Habana del Este (mm/h).

Duration (min)	T = 2	T = 5	T = 10	T = 25	T = 50	T = 100	T = 1000
10	86.2	103.7	114.9	129.6	142.1	152.4	188.3
20	70.3	85.1	94.5	106.7	117.3	125.9	156.2
30	59.7	72.7	80.9	91.5	100.7	108.2	134.7
40	52.2	63.8	71.1	80.6	88.7	95.5	119.1
50	46.5	57.0	63.6	72.2	79.6	85.8	107.2
60	42.0	51.7	57.8	65.7	72.5	78.1	97.8
90	32.9	40.9	45.8	52.2	57.7	62.3	78.4
120	27.3	34.1	38.3	43.8	48.5	52.4	66.2
180	20.7	26.1	29.4	33.7	37.5	40.5	51.4
360	12.5	16.0	18.2	21.0	23.4	25.4	32.5
720	7.3	9.6	11.0	12.8	14.3	15.6	20.1
1080	5.3	7.1	8.1	9.5	10.7	11.6	15.1
1440	4.3	5.7	6.6	7.7	8.7	9.4	12.3

To facilitate practical application in hydrological design, these values were fitted to the Sherman model. The fitted parameters for each return period, along with the coefficient of determination (R^2^), are shown below (Table 2):

**Table 2:** Sherman equation parameters for unified IDF curve.

Return Period (T)	a	B	c	R²
2 years	1473.46	24.47	0.8019	0.999
5 years	1603.82	24.36	0.7744	0.999
10 years	1693.30	24.15	0.7620	0.999
25 years	1820.77	23.91	0.7500	0.999
50 years	1947.71	23.90	0.7430	0.999
100 years	2064.81	24.01	0.7391	0.999
1000 years	2442.48	24.13	0.7260	0.999

**Fig 1:**
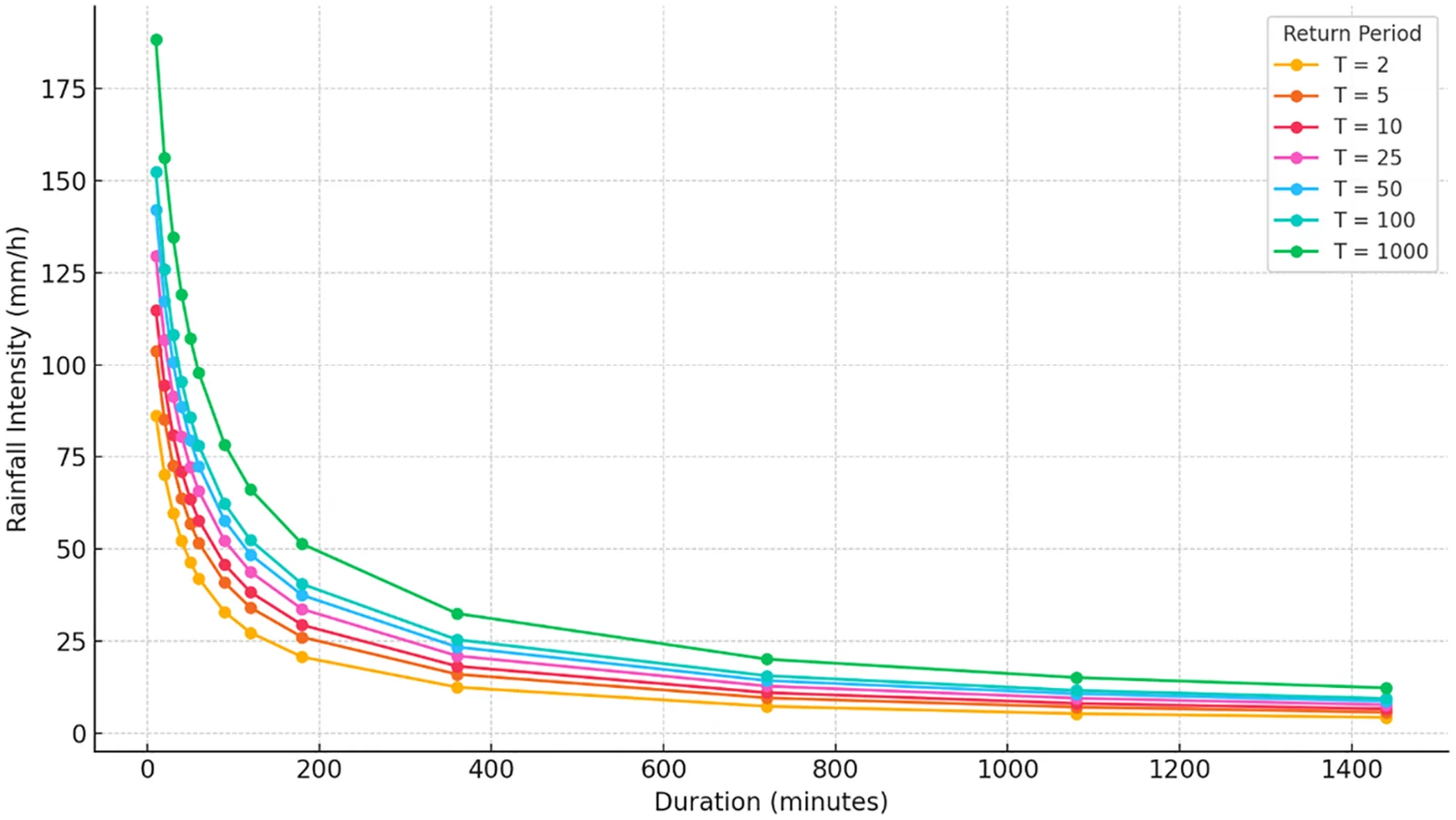
Final unified IDF curves. (linear scale).

To visually validate these results, Fig 1 displays the unified IDF curves across all return periods. The figure confirms the expected monotonic decay of intensity with duration and the clear separation between return periods, reflecting the increasing severity of rare events.

**Fig 2:**
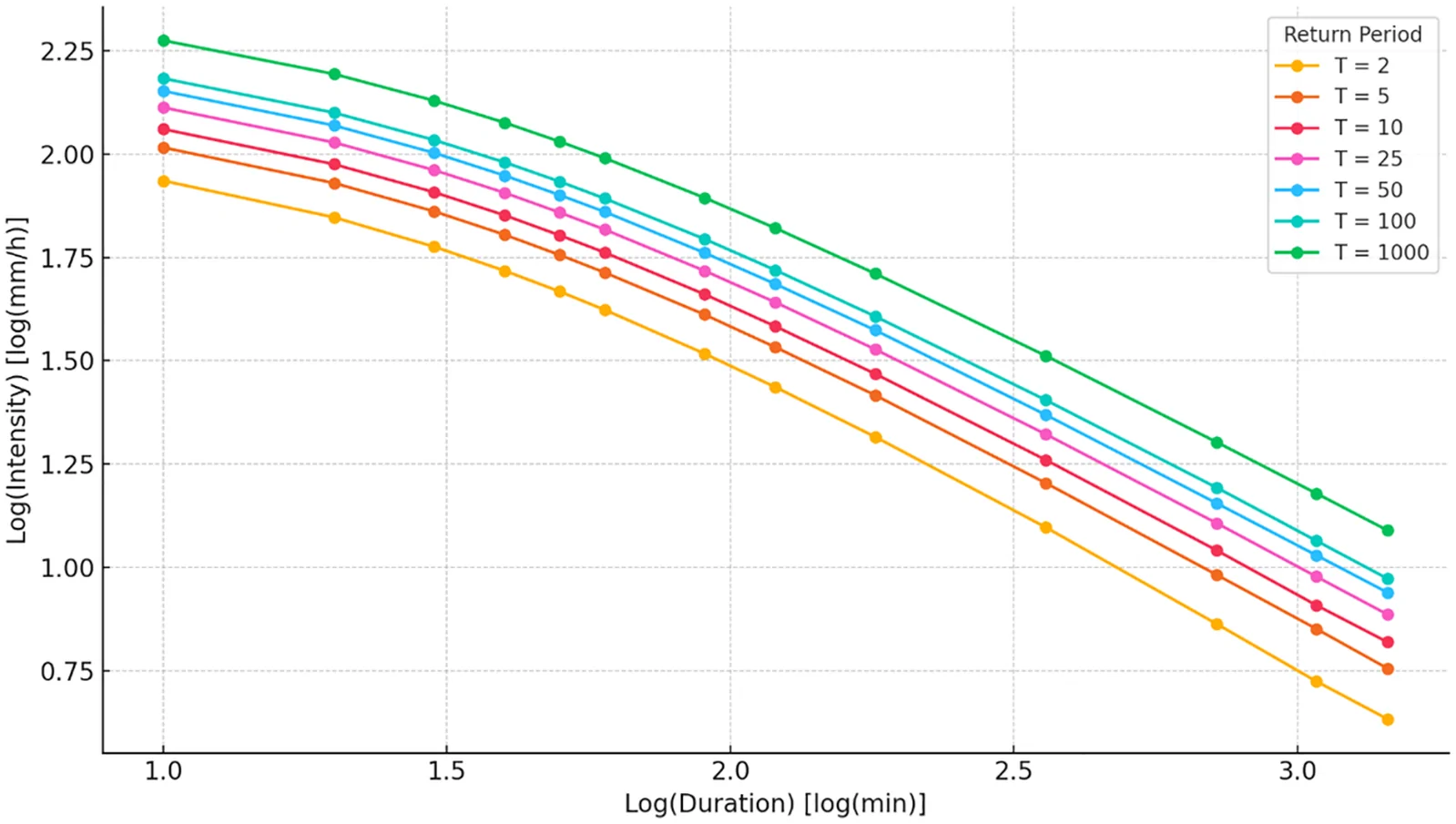
Final unified IDF curves (log–log scale). Plot of log(intensity) vs. log(duration), showing consistent linearity and excellent curve fitting across durations.

To assess the quality of the Sherman model fitting, Fig 2 presents the same data in log–log scale. The near-linear alignment of points in this space validates the empirical model structure and supports its applicability for extrapolation.

**Fig 3:**
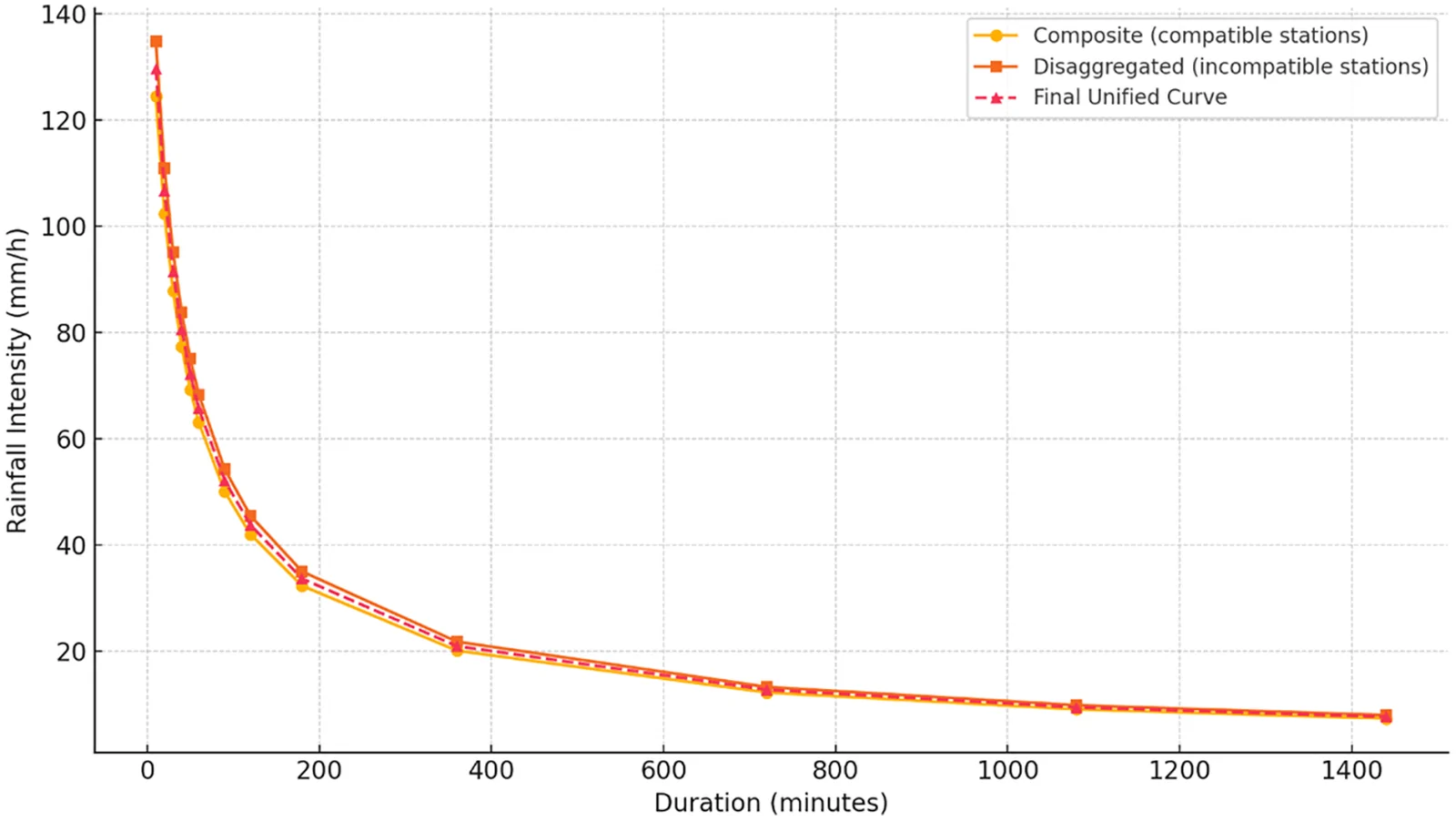
Comparison of composite, disaggregated, and final unified curves (T = 25 years). A comparative plot showing intensity vs duration for T = 25 years, highlighting the coherence between both methodological pathways and the final integrated result.

**Fig 4:**
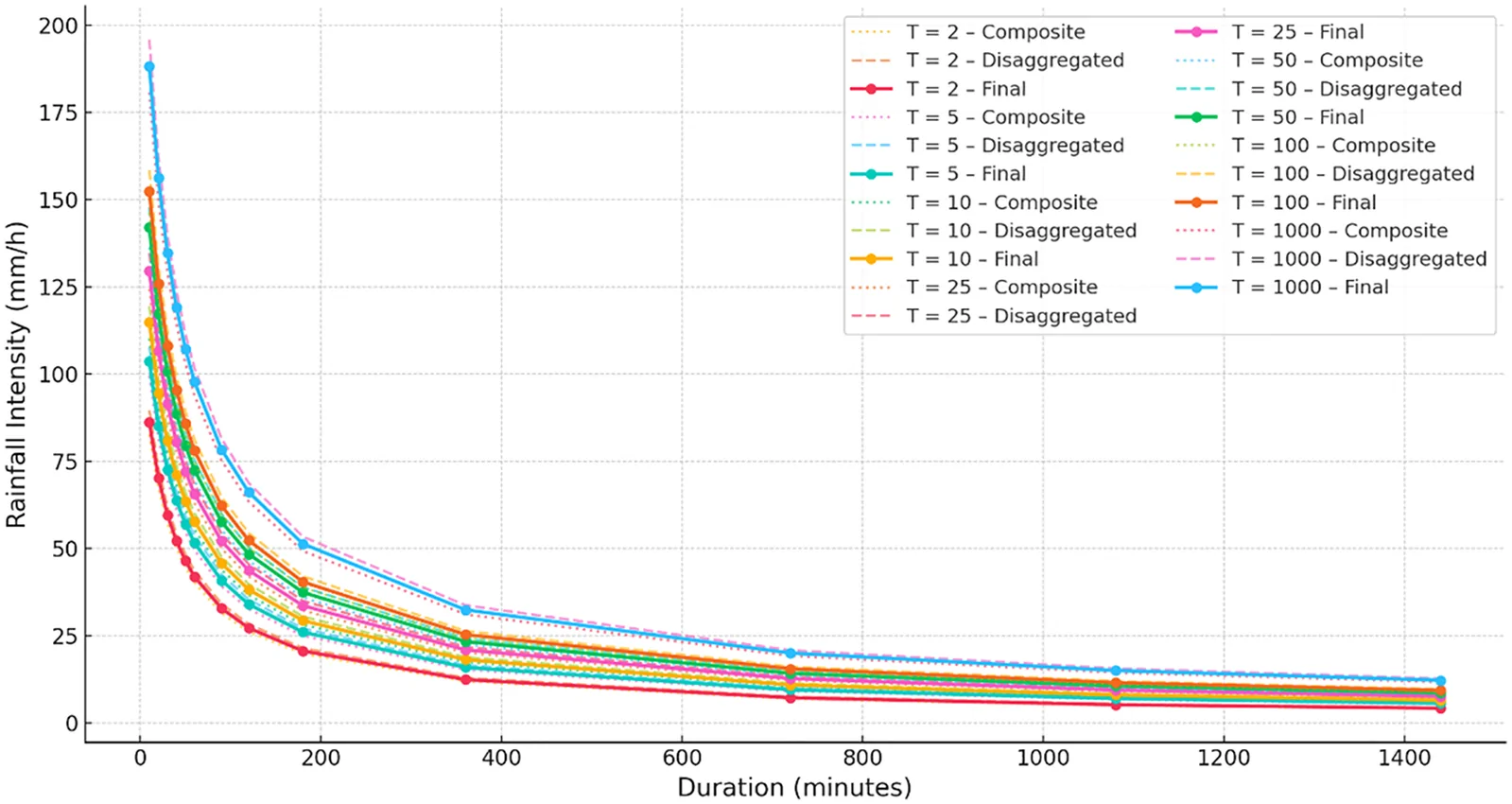
Composite vs. disaggregated vs. final IDF curves for all return periods. A multi-series plot displaying IDF curves for composite, disaggregated, and final methods across durations and all return periods. This figure illustrates the low deviation and structural convergence across estimation techniques.

Together, these visual and tabular results provide a robust and replicable IDF framework tailored to data-scarce urban regions such as Habana del Este. In addition, a comparative analysis was developed to evaluate the contributions of the composite and disaggregated datasets separately. As shown in Figs 3 and 4, the intensity–duration curve derived from the disaggregated group exhibits slightly higher values, while the composite curve is slightly lower. The final unified curve aligns between them, supporting the methodological choice of weighted integration. Notice that for all return periods there is a consistent structural relationship where final unified curves are framed between their respective composite and disaggregated counterparts. This symmetry strengthens confidence in the representativeness and internal consistency of the unified IDF formulation. These outputs provide a reliable and implementable design tool for hydrological engineering in Habana del Este and potentially similar data-limited urban regions.

## Discussion

The unified IDF curves developed for Habana del Este demonstrate the feasibility of reconstructing sub-daily rainfall intensity patterns from daily observations, even in the absence of long-term high-resolution data. The integration of pluviograph-based intensities with disaggregated pluviometric data through a compatibility and weighting scheme yields a regionalized model that is statistically robust, spatially representative, and operationally practical.

The near-linear behavior observed in the log–log plots (Fig 2) provides visual support for the appropriateness of the Sherman model. While the fitted curves do not exhibit perfect linearity, particularly at very short or very long durations, this is expected due to the structure of the Sherman equation, which includes a temporal translation parameter. The presence of the expression (t + b) in the denominator introduces a slight curvature in the log–log space, particularly where t∼b, which is consistent with the physical nature of precipitation decay and the empirical calibration approach.

Additionally, the comparison between the composite, disaggregated, and final unified curves (Figs 3 and 4) validates the methodological choice to combine stations with differing levels of compatibility through dual processing strategies. The consistently small deviations between the component curves and the unified result confirm that the integrated model successfully captures the central tendency of the local rainfall regime without introducing distortion from the disaggregation procedure. This is particularly relevant in urban tropical contexts, where intense short-duration storms can pose significant hydraulic challenges.

Despite the success of the approach, it is important to recognize its limitations. The disaggregation model assumes a uniform intra-day rainfall distribution pattern across all years and return levels, which may oversimplify the temporal variability of convective rainfall dynamics. This assumption may smooth the concentration of intense short-duration storms and, consequently, may lead to underestimation of peak rainfall intensities at the shortest durations, particularly for 10, 20, 30, and 60 min events. In practical terms, this could affect design rainfall estimates for small urban drainage structures, culverts, local stormwater systems, and other hydraulic works where short-duration peak intensities are particularly relevant. Furthermore, while the Gumbel distribution provided a good statistical fit, it may underestimate upper-tail behavior under changing climate conditions. The use of a 15-year daily rainfall record also represents a limitation for estimating very rare return periods, particularly the 1000-year return level. Therefore, the highest return-period estimates should be interpreted as extrapolated design values rather than direct empirical observations. Future work should address these limitations by calibrating the disaggregation parameter with longer local pluviograph records, radar-derived rainfall estimates, satellite precipitation products, or stochastic storm profiles representative of tropical convective rainfall.

Nevertheless, the methodology provides a defensible and replicable framework for generating IDF curves in data-scarce settings. Its ability to incorporate diverse datasets and maintain internal consistency makes it a promising tool for hydrological design, especially in regions where infrastructure planning must proceed in the absence of dense monitoring networks.

Future work could explore the integration of radar-derived rainfall estimates, regional climate model outputs, longer rainfall records, or non-stationary frequency analysis to better reflect long-term shifts in rainfall intensity patterns. In addition, future studies should calibrate the disaggregation parameter using local high-resolution rainfall records, when available, to improve the representation of short-duration convective rainfall peaks.

## Conclusions

This study developed and evaluated a practical framework for reconstructing intensity–duration–frequency (IDF) curves in data-scarce tropical environments, using Habana del Este, Cuba, as a case study. The main contribution lies in the integration of station compatibility screening, direct use of compatible pluviometric information, temporal disaggregation of non-compatible daily records, and weighted regional unification into a single reproducible workflow.

The results indicate that daily rainfall records, when combined with limited sub-daily reference information and appropriate consistency checks, can provide defensible IDF estimates for engineering design and urban hydrological planning. Although uncertainties remain, particularly regarding short-duration peak intensities and very rare return periods, the proposed approach offers a transparent alternative for regions where conventional pluviograph-based IDF development is not feasible.

The framework may be adapted to other tropical regions with similar data constraints, provided that quality-controlled daily maximum rainfall series, local or regional information on sub-daily rainfall structure, and station homogeneity and compatibility checks are available. Under these conditions, it can support preliminary or regional IDF curve development for hydraulic design, stormwater management, and climate resilience planning.

## Supporting information

S1 File Dataset used for IDF curve reconstruction. Excel file containing hydrometeorological data, processed rainfall records, intermediate calculations, Gumbel distribution estimates, temporal disaggregation results, Sherman equation fitting parameters, and supporting outputs used for the reconstruction and validation of the IDF curves.
(XLSX)
