# Supplementary material for: Reconstructing IDF curves from daily rainfall records in data-scarce regions: A statistical method based on temporal disaggregation and gumbel modeling
Source: PLoS One. 2026 Jul 9;21(7):e0351841. doi: 10.1371/journal.pone.0351841 (PMC13488912; doi:10.1371/journal.pone.0351841)
Supplement: Supplementary file 1 [file pone.0351841.s003.docx]

Response to Reviewers' Comments.

*Ms. Ref. No.: PONE-D-25-42783*

*Title: Reconstructing IDF Curves from Daily Rainfall Records in Data-Scarce Regions: A Statistical Method Based on Temporal Disaggregation and Gumbel Modeling*

We sincerely thank the Academic Editor and both reviewers for their careful evaluation of our manuscript and for their constructive comments. We appreciate the positive assessment of the technical soundness, statistical analysis, relevance, and overall structure of the study. The reviewers’ observations were very helpful in improving the clarity, transparency, reproducibility, and practical framing of the manuscript.

In the revised version, we have addressed all comments point by point. Specifically, we clarified the novelty and specific contribution of the study, corrected the inconsistency regarding the period of analysis, expanded the justification for the temporal disaggregation parameter, strengthened the discussion of limitations, improved the explanation of the practical decision-making value of the proposed method, and revised the Data Availability Statement.

Detailed responses to each comment are provided below.

### **Reviewer #1**

**The manuscript is generally well written, presenting a clear research problem, a sound methodological framework, and a comprehensive discussion of results. The authors address an important issue, the development of intensity, duration, frequency (IDF) curves in regions where the lack of sub-daily rainfall data limits climate-resilient infrastructure planning and development. The topic is timely, relevant, and well aligned with hydrological and climate adaptation research.Overall, the study contributes valuable insights and presents a practical methodology for IDF curve reconstruction in data-scarce environments. However, I suggest a few minor revisions that could further enhance the clarity and scientific rigor of the paper.**

1. **Clarify the novelty and specific contribution: While the manuscript demonstrates methodological rigor, the novelty of the work is not explicitly stated. The introduction and abstract should clearly highlight what differentiates this approach from existing studies that also apply temporal disaggregation and Gumbel modeling. Therefore, if possible, add a concise statement clarifying the unique methodological advancement, regional contribution, or integration framework introduced in this paper.**

We thank the reviewer for this important observation. We agree that the novelty and specific contribution of the manuscript needed to be stated more explicitly. In the revised version, we clarified that the contribution of the study does not rely solely on the independent use of temporal disaggregation or Gumbel frequency analysis, which are established methods, but on their integration into a practical and reproducible framework for data-scarce tropical urban regions. Specifically, we revised the Abstract and Introduction to emphasize that the study combines: (i) compatibility assessment between pluviometric stations and a reference pluviograph, (ii) direct integration of hydrologically compatible stations, (iii) temporal disaggregation of non-compatible daily rainfall records, and (iv) weighted regional unification of the resulting IDF parameters into a single representative model. This integrated workflow represents the main methodological contribution of the paper and provides a practical approach for reconstructing IDF curves in regions where sub-daily rainfall records are limited.

**The following changes were made in the abstract:**

***Original:*** *Urban regions in the tropics often face challenges in hydrological design due to the lack of high-resolution rainfall data. This study presents a method for generating intensity, duration, frequency (IDF) curves using only daily rainfall records, applied to the case of Habana del Este, Cuba. Daily maximum rainfall data from eight pluviometric stations (2010–2024) were combined with subdaily observations from a reference pluviograph. Two strategies were used: direct integration for compatible stations and temporal disaggregation for incompatible ones. Rainfall intensities for return periods between 2 and 1000 years were estimated using Gumbel frequency analysis and fitted to the Sherman model through nonlinear regression. The resulting IDF curves were unified into a single regional model using a weighted average of the fitting parameters. The final curves showed excellent agreement across durations and return levels (R² > 0.998), with strong internal consistency between estimation methods. Validation using linear and log log plots confirmed the robustness of the approach. This method provides a practical and statistically sound solution for IDF curve development in data scarce environments, offering valuable support for infrastructure planning and climate resilience in tropical urban settings.*

*🡪*

***Modification:*** *Urban regions in the tropics often face challenges in hydrological design due to the lack of high-resolution rainfall data. This study presents a method for generating intensity, duration, frequency (IDF) curves using only daily rainfall records, applied to the case of Habana del Este, Cuba. Daily maximum rainfall data from eight pluviometric stations (2010–2024) were combined with subdaily observations from a reference pluviograph. Two strategies were used: direct integration for compatible stations and temporal disaggregation for incompatible ones. Rainfall intensities for return periods between 2 and 1000 years were estimated using Gumbel frequency analysis and fitted to the Sherman model through nonlinear regression. The resulting IDF curves were unified into a single regional model using a weighted average of the fitting parameters. The main contribution of this study is an integrated reconstruction framework that links station compatibility screening, selective use of observed subdaily rainfall structure, disaggregation of non-compatible daily records, and weighted regional unification within a single reproducible workflow. The final curves showed excellent agreement across durations and return levels (R² > 0.998), with strong internal consistency between estimation methods. Validation using linear and log log plots confirmed the robustness of the approach. This method provides a practical and statistically sound solution for IDF curve development in data scarce tropical regions, offering direct support for infrastructure planning, hydraulic design, and climate resilience where subdaily rainfall observations are limited.*

2. **Dataset inconsistency for period of analysis: There appears to be an inconsistency in the dataset period mentioned: Line 92 (Introduction) refers to a 35-year dataset (presumably 1990-2024). Line 162 (Methods) and Abstract (line 17) mention data for 2010-2024 (15 years). Please clarify the actual period of record used and correct these statements if needed throughout the text. If data reconstruction or extension was involved, specify how earlier data were obtained or estimated and whether the period length was sufficient for robust frequency analysis.**

We thank the reviewer for identifying this inconsistency. We confirm that the actual period of analysis used in this study was 2010–2024, corresponding to a 15-year record of daily maximum rainfall data from the selected pluviometric stations. The reference to a 35 year dataset in the Introduction was an editorial error and has been corrected throughout the revised manuscript. No extension of the dataset back to 1990 was performed. The only reconstruction applied in the study was limited to missing annual maximum values within the 2010–2024 period for one pluviometric station, using the mean ratio method based on homogeneous reference stations. This reconstruction procedure has now been clarified in the Methods section.

We also added a brief clarification regarding the adequacy and limitations of the 15-year record. While longer records are generally preferable for extreme rainfall frequency analysis, the 2010-2024 period was considered acceptable for the objective of this study because it provides a continuous and homogeneous dataset within the available official records, and because the proposed framework is intended for data-scarce regions where long-term sub-daily rainfall series are not available. Nevertheless, we now explicitly acknowledge this record length as a limitation, particularly for estimating very rare return periods such as 1000 years.

The introduction was modified as follows:

***Original:****Accordingly, this study proposes and validates a method to reconstruct IDF curves exclusively from daily rainfall data. The approach combines temporal disaggregation with Gumbel distribution fitting, requiring only daily maxima and standard statistical tools. It was applied to a 35-year dataset from Habana del Este, a coastal municipality in Cuba, and is intended for broader use in other data-scarce regions where sub-daily observations are lacking. The proposed methodology emphasizes accessibility, simplicity, and replicability in contexts where resource constraints make traditional pluviograph-based analyses impractical.*

*🡪*

***Modification:****Accordingly, this study proposes and validates a method to reconstruct IDF curves from daily rainfall data. The approach combines compatibility screening, temporal disaggregation, Gumbel frequency analysis, Sherman model fitting, and weighted regional unification. It was applied to a 15-year dataset of daily maximum rainfall records from Habana del Este, Cuba, covering the period 2010–2024.*

The methodology section was improved as follows:

***Original****: Daily maximum rainfall data for the period 2010–2024 were obtained from the Sistema de Gestión Integral del Agua (SGIA), Cuba’s official hydrometeorological platform. Initially, ten pluviometric stations located within or near Habana del Este were considered. A preliminary quality check eliminated two stations with insufficient temporal coverage or unreliable records. The remaining eight stations were subjected to homogeneity analysis using the coefficient of variation (CV), comparing the relative dispersion of each series against the group mean. Seven stations met the ±15% homogeneity criterion; the eighth, though initially excluded due to anomalous dispersion, was later reconstructed.*

*🡪*

***Modification:*** *Daily maximum rainfall data for the period 2010–2024 were obtained from the Sistema de Gestión Integral del Agua (SGIA), Cuba’s official hydrometeorological platform. No extension of the dataset prior to 2010 was performed. Initially, ten pluviometric stations located within or near Habana del Este were considered. A preliminary quality check eliminated two stations with insufficient temporal coverage or unreliable records. The remaining eight stations were subjected to homogeneity analysis using the coefficient of variation (CV), comparing the relative dispersion of each series against the group mean. Seven stations met the ±15% homogeneity criterion; the eighth station, which initially showed anomalous dispersion due to incomplete annual maximum values within the 2010–2024 period, was reconstructed using the mean ratio method and subsequently reevaluated for homogeneity.*

The following sentence was added to the methodology as well:

*Therefore, the final frequency analysis was based exclusively on annual maximum rainfall series within the 2010–2024 period.*

3. **Justification for parameter k= 0.45. In line 136, the value of k= 0.45 was used for exponential disaggregation. While this choice may be empirically reasonable, the manuscript would benefit from a brief justification or reference for adopting this specific value. Clarify whether k= 0.45 was derived from calibration with local pluviograph data, from literature specific to tropical climates, or adopted as a standard assumption. This will improve transparency and reproducibility.**

We thank the reviewer for this helpful comment. We agree that selecting the disaggregation parameter k = 0.45 required clearer justification. In the revised manuscript, we clarified that k = 0.45 was neither derived from a full local calibration against a complete pluviograph archive nor treated as a universal constant. Instead, it was adopted as an empirical disaggregation exponent suitable for humid tropical rainfall regimes, particularly those influenced by convective and convective stratiform events.

We have revised the Theory and Materials and Methods sections to clarify that this parameter was selected based on hydrological guidance and prior regional applications in tropical rainfall disaggregation. We also clarified that its use was evaluated through the internal consistency of the resulting IDF curves, including smoothness, monotonic decay with increasing duration, and agreement between the disaggregated and unified IDF estimates.

The theory section was improved and modified as follows:

***Original:*** *Here, k controls the rate at which rainfall accumulates during a storm. Based on empirical calibration, k=0.45 was adopted in this work. This function allows partitioning daily rainfall totals h24 into shorter durations:*

*🡪*

***Modification:*** *Here, k controls the rate at which the daily rainfall depth is redistributed into shorter durations. In this study, k = 0.45 was adopted as an empirical disaggregation exponent suitable for humid tropical rainfall regimes, particularly those influenced by convective and convective stratiform storm structures. This value was not treated as a universal constant or as the result of a full local pluviograph calibration. Rather, it was selected based on hydrological guidance and previous regional applications for tropical rainfall disaggregation, and its use was subsequently evaluated by assessing the internal consistency of the resulting IDF curves. This function allows partitioning daily rainfall totals h24 into shorter durations:*

The Methodology section was modified as well:

***Original:****Stations found incompatible with the pluviograph were processed separately using a temporal disaggregation procedure. The adopted model, based on an exponential decay function calibrated with k=0.45, redistributed each 24-hour precipitation total into shorter durations, preserving volume and reflecting typical storm structures in tropical climates.*

*🡪*

***Modification:*** *Stations found incompatible with the pluviograph were processed separately using a temporal disaggregation procedure. The adopted model used an empirical exponent of k = 0.45 to redistribute each 24-hour precipitation total into shorter durations while preserving the daily rainfall volume. This value was selected because it is consistent with humid tropical rainfall regimes dominated by convective and convective stratiform events, as reported in regional hydrological guidance and applied disaggregation procedures. The parameter was not derived from a full calibration against local pluviograph records; therefore, its use was assessed by examining the smoothness, monotonic behavior, and internal agreement of the resulting IDF curves.*

4. **Discussion of limitations and implications. In line 282, the authors acknowledge the assumption of a uniform intra-day rainfall distribution as a simplification. This is an important limitation, but its quantitative or conceptual impact on results is not sufficiently discussed. Please elaborate on how this assumption may influence the derived IDF curves, for example, whether it could lead to underestimation of peak intensities at shorter durations or affect design rainfall estimates. Additionally, suggest potential ways to address this limitation in future work.**

We expanded the limitations paragraph to clarify that the uniform intra-day rainfall distribution assumption may smooth intense convective rainfall peaks and lead to underestimation of short-duration intensities, particularly for 10, 20, 30, and 60 min events. We also clarified that this may affect design rainfall estimates for small urban drainage structures, culverts, and local stormwater systems. Finally, we added specific future work recommendations, including calibration with longer local pluviograph records, radar rainfall estimates, satellite precipitation products, and stochastic storm profiles. The discussion was improved as follows:

***Original:*** *Despite the success of the approach, it is important to recognize its limitations. The disaggregation model assumes a uniform intra-day rainfall distribution pattern across all years and return levels, which may oversimplify the temporal variability of convective rainfall dynamics. This assumption may smooth the concentration of intense short-duration storms and, consequently, may lead to underestimation of peak rainfall intensities at the shortest durations. In practical terms, this could affect design rainfall estimates for small urban drainage structures, where short-duration peak intensities are particularly relevant. Furthermore, while the Gumbel distribution provided a good statistical fit, it may underestimate upper-tail behavior under changing climate conditions. The use of a 15-year daily rainfall record also represents a limitation for estimating very rare return periods, particularly the 1000-year return level. Therefore, the highest return-period estimates should be interpreted as extrapolated design values rather than direct empirical observations.*

*🡪*

***Modification:*** *Despite the success of the approach, it is important to recognize its limitations. The disaggregation model assumes a uniform intra-day rainfall distribution pattern across all years and return levels, which may oversimplify the temporal variability of convective rainfall dynamics. This assumption may smooth the concentration of intense short-duration storms and, consequently, may lead to underestimation of peak rainfall intensities at the shortest durations, particularly for 10, 20, 30, and 60 min events. In practical terms, this could affect design rainfall estimates for small urban drainage structures, culverts, local stormwater systems, and other hydraulic works where short-duration peak intensities are particularly relevant. Furthermore, while the Gumbel distribution provided a good statistical fit, it may underestimate upper-tail behavior under changing climate conditions. The use of a 15-year daily rainfall record also represents a limitation for estimating very rare return periods, particularly the 1000-year return level. Therefore, the highest return-period estimates should be interpreted as extrapolated design values rather than direct empirical observations. Future work should address these limitations by calibrating the disaggregation parameter with longer local pluviograph records, radar-derived rainfall estimates, satellite precipitation products, or stochastic storm profiles representative of tropical convective rainfall.*

5. **It may strengthen the paper to include a short practical discussion on how this reconstructed IDF method could be applied or adapted to other tropical regions facing similar data constraints. A few sentences in the conclusion on the transferability and scalability of the approach would enhance the paper’s impact.Overall Recommendation: The manuscript presents a valuable and well-structured contribution to rainfall frequency analysis in data-scarce regions. After minor clarifications and justifications as noted above, it would be suitable for publication.**

We thank the reviewer for this valuable suggestion. We agree that the manuscript would benefit from a clearer explanation of the transferability and scalability of the proposed method. In the revised Conclusion, we added a short practical discussion explaining how the framework could be adapted to other tropical regions where daily rainfall records are available but long-term sub-daily observations are limited.

We clarified that transferability depends on the availability of quality-controlled daily maximum rainfall series, a reference source of sub-daily rainfall structure or regional disaggregation guidance, and local verification of station homogeneity and compatibility. We also emphasized that, under these conditions, the method can support the development of preliminary or regional IDF curves for hydraulic design, stormwater planning, and climate resilience assessments in data-scarce urban and peri-urban environments.

We improve the Conclusion was revised to discuss the transferability and scalability of the proposed IDF reconstruction framework to other tropical data-scarce regions.

### **Reviewer #2**

**This manuscript has written well and I recommend for the publication with major revision:
(i) how this study is different than other studies with similar analysis
(ii) the research gap was not clearly written in the introduction section
(iii) One thing is missing in this paper how the entire analysis helps in the decision-making process.**

We thank the reviewer for the positive assessment of the manuscript and for recommending publication after major revision. We also appreciate the three important points raised, as they helped us strengthen the positioning, research gap, and practical relevance of the study.

Regarding point (i), we revised the Abstract and Introduction to clarify how this study differs from previous studies using similar tools such as temporal disaggregation and Gumbel frequency analysis. We now emphasize that the contribution of the study is not the isolated use of these methods, but their integration into a reproducible IDF reconstruction framework that combines station compatibility screening, direct incorporation of hydrologically compatible stations, temporal disaggregation of non-compatible daily records, and weighted regional unification into a single representative model.

Regarding point (ii), we revised the Introduction to state the research gap more explicitly. We clarified that, although several studies have estimated IDF curves from daily rainfall records, fewer studies provide a transparent operational framework for deciding when daily pluviometric stations can be directly integrated with pluviograph-derived curves and when they require independent disaggregation before regional unification. This gap is particularly relevant in tropical urban regions where sub-daily rainfall records are limited but engineering design decisions still require locally derived IDF estimates.

Regarding point (iii), we expanded the practical discussion of the study’s implications. In the revised Discussion and Conclusion, we clarified how the reconstructed IDF curves can support decision-making in hydraulic design, stormwater management, flood risk reduction, infrastructure prioritization, and climate resilience planning. We also explained that the method can help engineers and local authorities develop preliminary or regional design rainfall estimates in areas where conventional pluviograph-based IDF analysis is not feasible.

**Thus, t**he Abstract and Introduction were revised to clarify the novelty and research gap. The Discussion and Conclusion were revised to strengthen the practical decision-making implications, transferability, and scalability of the proposed IDF reconstruction framework.
