# Supplementary material for: Reconstructing IDF curves from daily rainfall records in data-scarce regions: A statistical method based on temporal disaggregation and gumbel modeling
Source: PLoS One. 2026 Jul 9;21(7):e0351841. doi: 10.1371/journal.pone.0351841 (PMC13488912; doi:10.1371/journal.pone.0351841)
Supplement: Peer review history

# Peer review: Decision Letter 0

**DOI:** 10.1371/journal.pone.0351841.r001

**Authors:** Nitin Bassi

20 Nov 2025

PONE-D-25-42783

Reconstructing IDF Curves from Daily Rainfall Records in Data-Scarce Regions: A Statistical Method Based on Temporal Disaggregation and Gumbel Modeling

PLOS ONE

Dear Dr. Molino,

Thank you for submitting your manuscript to PLOS ONE. After careful consideration, we feel that it has merit but does not fully meet PLOS ONE’s publication criteria as it currently stands. Therefore, we invite you to submit a revised version of the manuscript that addresses the points raised during the review process.

Please submit your revised manuscript by Jan 04 2026 11:59PM. If you will need more time than this to complete your revisions, please reply to this message or contact the journal office at plosone@plos.org. When you're ready to submit your revision, log on to https://www.editorialmanager.com/pone/ and select the 'Submissions Needing Revision' folder to locate your manuscript file.

Please include the following items when submitting your revised manuscript:

- A rebuttal letter that responds to each point raised by the academic editor and reviewer(s). You should upload this letter as a separate file labeled 'Response to Reviewers'.
- A marked-up copy of your manuscript that highlights changes made to the original version. You should upload this as a separate file labeled 'Revised Manuscript with Track Changes'.
- An unmarked version of your revised paper without tracked changes. You should upload this as a separate file labeled 'Manuscript'.

If you would like to make changes to your financial disclosure, please include your updated statement in your cover letter. Guidelines for resubmitting your figure files are available below the reviewer comments at the end of this letter.

If applicable, we recommend that you deposit your laboratory protocols in protocols.io to enhance the reproducibility of your results. Protocols.io assigns your protocol its own identifier (DOI) so that it can be cited independently in the future. For instructions see: https://journals.plos.org/plosone/s/submission-guidelines#loc-laboratory-protocols. Additionally, PLOS ONE offers an option for publishing peer-reviewed Lab Protocol articles, which describe protocols hosted on protocols.io. Read more information on sharing protocols at https://plos.org/protocols?utm_medium=editorial-email&utm_source=authorletters&utm_campaign=protocols.

We look forward to receiving your revised manuscript.

Kind regards,

Nitin Bassi

Academic Editor

PLOS ONE

Journal Requirements:

When submitting your revision, we need you to address these additional requirements.

1. Please ensure that your manuscript meets PLOS ONE's style requirements, including those for file naming. The PLOS ONE style templates can be found at

https://journals.plos.org/plosone/s/file?id=wjVg/PLOSOne_formatting_sample_main_body.pdf and

https://journals.plos.org/plosone/s/file?id=ba62/PLOSOne_formatting_sample_title_authors_affiliations.pdf

If the reviewer comments include a recommendation to cite specific previously published works, please review and evaluate these publications to determine whether they are relevant and should be cited. There is no requirement to cite these works unless the editor has indicated otherwise.

[Note: HTML markup is below. Please do not edit.]

Reviewers' comments:

Reviewer's Responses to Questions

**Comments to the Author**

1. Is the manuscript technically sound, and do the data support the conclusions?

The manuscript must describe a technically sound piece of scientific research with data that supports the conclusions. Experiments must have been conducted rigorously, with appropriate controls, replication, and sample sizes. The conclusions must be drawn appropriately based on the data presented.

Reviewer #1: Yes

Reviewer #2: Yes

**********

2. Has the statistical analysis been performed appropriately and rigorously?

Reviewer #1: Yes

Reviewer #2: Yes

**********

3. Have the authors made all data underlying the findings in their manuscript fully available?

The PLOS Data policy requires authors to make all data underlying the findings described in their manuscript fully available without restriction, with rare exception (please refer to the Data Availability Statement in the manuscript PDF file). The data should be provided as part of the manuscript or its supporting information, or deposited to a public repository. For example, in addition to summary statistics, the data points behind means, medians and variance measures should be available. If there are restrictions on publicly sharing data—e.g. participant privacy or use of data from a third party—those must be specified.

Reviewer #1: Yes

Reviewer #2: No

**********

4. Is the manuscript presented in an intelligible fashion and written in standard English?

PLOS ONE does not copyedit accepted manuscripts, so the language in submitted articles must be clear, correct, and unambiguous. Any typographical or grammatical errors should be corrected at revision, so please note any specific errors here.

Reviewer #1: Yes

Reviewer #2: Yes

**********

5. Review Comments to the Author

Please use the space provided to explain your answers to the questions above. You may also include additional comments for the author, including concerns about dual publication, research ethics, or publication ethics. (Please upload your review as an attachment if it exceeds 20,000 characters)

Reviewer #1: The manuscript is generally well written, presenting a clear research problem, a sound methodological framework, and a comprehensive discussion of results. The authors address an important issue, the development of intensity, duration, frequency (IDF) curves in regions where the lack of sub-daily rainfall data limits climate-resilient infrastructure planning and development. The topic is timely, relevant, and well aligned with hydrological and climate adaptation research.

Overall, the study contributes valuable insights and presents a practical methodology for IDF curve reconstruction in data-scarce environments. However, I suggest a few minor revisions that could further enhance the clarity and scientific rigor of the paper.

1. Clarify the novelty and specific contribution: While the manuscript demonstrates methodological rigor, the novelty of the work is not explicitly stated. The introduction and abstract should clearly highlight what differentiates this approach from existing studies that also apply temporal disaggregation and Gumbel modeling. Therefore, if possible, add a concise statement clarifying the unique methodological advancement, regional contribution, or integration framework introduced in this paper.

2. Dataset inconsistency for period of analysis: There appears to be an inconsistency in the dataset period mentioned: Line 92 (Introduction) refers to a 35-year dataset (presumably 1990-2024). Line 162 (Methods) and Abstract (line 17) mention data for 2010-2024 (15 years). Please clarify the actual period of record used and correct these statements if needed throughout the text. If data reconstruction or extension was involved, specify how earlier data were obtained or estimated and whether the period length was sufficient for robust frequency analysis.

3. Justification for parameter k= 0.45. In line 136, the value of k= 0.45 was used for exponential disaggregation. While this choice may be empirically reasonable, the manuscript would benefit from a brief justification or reference for adopting this specific value. Clarify whether k= 0.45 was derived from calibration with local pluviograph data, from literature specific to tropical climates, or adopted as a standard assumption. This will improve transparency and reproducibility.

4. Discussion of limitations and implications. In line 282, the authors acknowledge the assumption of a uniform intra-day rainfall distribution as a simplification. This is an important limitation, but its quantitative or conceptual impact on results is not sufficiently discussed. Please elaborate on how this assumption may influence the derived IDF curves, for example, whether it could lead to underestimation of peak intensities at shorter durations or affect design rainfall estimates. Additionally, suggest potential ways to address this limitation in future work.

5. It may strengthen the paper to include a short practical discussion on how this reconstructed IDF method could be applied or adapted to other tropical regions facing similar data constraints. A few sentences in the conclusion on the transferability and scalability of the approach would enhance the paper’s impact.

Overall Recommendation: The manuscript presents a valuable and well-structured contribution to rainfall frequency analysis in data-scarce regions. After minor clarifications and justifications as noted above, it would be suitable for publication.

Reviewer #2: This manuscript has written well and I recommend for the publication with major revision:

(i) how this study is different than other studies with similar analaysis

(ii) the research gap was not clearly written in the introduction section

(iii) One thing is missing in this paper how the entire analysis helps in the decision making process.

**********

6. PLOS authors have the option to publish the peer review history of their article (what does this mean?). If published, this will include your full peer review and any attached files.

If you choose “no”, your identity will remain anonymous but your review may still be made public.

**Do you want your identity to be public for this peer review?**  For information about this choice, including consent withdrawal, please see our Privacy Policy.

Reviewer #1: No

Reviewer #2: No

**********

[NOTE: If reviewer comments were submitted as an attachment file, they will be attached to this email and accessible via the submission site. Please log into your account, locate the manuscript record, and check for the action link "View Attachments". If this link does not appear, there are no attachment files.]

To ensure your figures meet our technical requirements, please review our figure guidelines: https://journals.plos.org/plosone/s/figures

You may also use PLOS’s free figure tool, NAAS, to help you prepare publication quality figures: https://journals.plos.org/plosone/s/figures#loc-tools-for-figure-preparation.

NAAS will assess whether your figures meet our technical requirements by comparing each figure against our figure specifications.

# Author comment: Author response to Decision Letter 1

**DOI:** 10.1371/journal.pone.0351841.r002

27 May 2026

Response to Reviewers' Comments.

Ms. Ref. No.: PONE-D-25-42783

Title: Reconstructing IDF Curves from Daily Rainfall Records in Data-Scarce Regions: A Statistical Method Based on Temporal Disaggregation and Gumbel Modeling

We sincerely thank the Academic Editor and both reviewers for their careful evaluation of our manuscript and for their constructive comments. We appreciate the positive assessment of the technical soundness, statistical analysis, relevance, and overall structure of the study. The reviewers’ observations were very helpful in improving the clarity, transparency, reproducibility, and practical framing of the manuscript.

In the revised version, we have addressed all comments point by point. Specifically, we clarified the novelty and specific contribution of the study, corrected the inconsistency regarding the period of analysis, expanded the justification for the temporal disaggregation parameter, strengthened the discussion of limitations, improved the explanation of the practical decision-making value of the proposed method, and revised the Data Availability Statement.

Detailed responses to each comment are provided below.

Reviewer #1

The manuscript is generally well written, presenting a clear research problem, a sound methodological framework, and a comprehensive discussion of results. The authors address an important issue, the development of intensity, duration, frequency (IDF) curves in regions where the lack of sub-daily rainfall data limits climate-resilient infrastructure planning and development. The topic is timely, relevant, and well aligned with hydrological and climate adaptation research.Overall, the study contributes valuable insights and presents a practical methodology for IDF curve reconstruction in data-scarce environments. However, I suggest a few minor revisions that could further enhance the clarity and scientific rigor of the paper.

1. Clarify the novelty and specific contribution: While the manuscript demonstrates methodological rigor, the novelty of the work is not explicitly stated. The introduction and abstract should clearly highlight what differentiates this approach from existing studies that also apply temporal disaggregation and Gumbel modeling. Therefore, if possible, add a concise statement clarifying the unique methodological advancement, regional contribution, or integration framework introduced in this paper.

We thank the reviewer for this important observation. We agree that the novelty and specific contribution of the manuscript needed to be stated more explicitly. In the revised version, we clarified that the contribution of the study does not rely solely on the independent use of temporal disaggregation or Gumbel frequency analysis, which are established methods, but on their integration into a practical and reproducible framework for data-scarce tropical urban regions. Specifically, we revised the Abstract and Introduction to emphasize that the study combines: (i) compatibility assessment between pluviometric stations and a reference pluviograph, (ii) direct integration of hydrologically compatible stations, (iii) temporal disaggregation of non-compatible daily rainfall records, and (iv) weighted regional unification of the resulting IDF parameters into a single representative model. This integrated workflow represents the main methodological contribution of the paper and provides a practical approach for reconstructing IDF curves in regions where sub-daily rainfall records are limited.

The following changes were made in the abstract:

Original: Urban regions in the tropics often face challenges in hydrological design due to the lack of high-resolution rainfall data. This study presents a method for generating intensity, duration, frequency (IDF) curves using only daily rainfall records, applied to the case of Habana del Este, Cuba. Daily maximum rainfall data from eight pluviometric stations (2010–2024) were combined with subdaily observations from a reference pluviograph. Two strategies were used: direct integration for compatible stations and temporal disaggregation for incompatible ones. Rainfall intensities for return periods between 2 and 1000 years were estimated using Gumbel frequency analysis and fitted to the Sherman model through nonlinear regression. The resulting IDF curves were unified into a single regional model using a weighted average of the fitting parameters. The final curves showed excellent agreement across durations and return levels (R² > 0.998), with strong internal consistency between estimation methods. Validation using linear and log log plots confirmed the robustness of the approach. This method provides a practical and statistically sound solution for IDF curve development in data scarce environments, offering valuable support for infrastructure planning and climate resilience in tropical urban settings.

Modification: Urban regions in the tropics often face challenges in hydrological design due to the lack of high-resolution rainfall data. This study presents a method for generating intensity, duration, frequency (IDF) curves using only daily rainfall records, applied to the case of Habana del Este, Cuba. Daily maximum rainfall data from eight pluviometric stations (2010–2024) were combined with subdaily observations from a reference pluviograph. Two strategies were used: direct integration for compatible stations and temporal disaggregation for incompatible ones. Rainfall intensities for return periods between 2 and 1000 years were estimated using Gumbel frequency analysis and fitted to the Sherman model through nonlinear regression. The resulting IDF curves were unified into a single regional model using a weighted average of the fitting parameters. The main contribution of this study is an integrated reconstruction framework that links station compatibility screening, selective use of observed subdaily rainfall structure, disaggregation of non-compatible daily records, and weighted regional unification within a single reproducible workflow. The final curves showed excellent agreement across durations and return levels (R² > 0.998), with strong internal consistency between estimation methods. Validation using linear and log log plots confirmed the robustness of the approach. This method provides a practical and statistically sound solution for IDF curve development in data scarce tropical regions, offering direct support for infrastructure planning, hydraulic design, and climate resilience where subdaily rainfall observations are limited.

2. Dataset inconsistency for period of analysis: There appears to be an inconsistency in the dataset period mentioned: Line 92 (Introduction) refers to a 35-year dataset (presumably 1990-2024). Line 162 (Methods) and Abstract (line 17) mention data for 2010-2024 (15 years). Please clarify the actual period of record used and correct these statements if needed throughout the text. If data reconstruction or extension was involved, specify how earlier data were obtained or estimated and whether the period length was sufficient for robust frequency analysis.

We thank the reviewer for identifying this inconsistency. We confirm that the actual period of analysis used in this study was 2010–2024, corresponding to a 15-year record of daily maximum rainfall data from the selected pluviometric stations. The reference to a 35 year dataset in the Introduction was an editorial error and has been corrected throughout the revised manuscript. No extension of the dataset back to 1990 was performed. The only reconstruction applied in the study was limited to missing annual maximum values within the 2010–2024 period for one pluviometric station, using the mean ratio method based on homogeneous reference stations. This reconstruction procedure has now been clarified in the Methods section.

We also added a brief clarification regarding the adequacy and limitations of the 15-year record. While longer records are generally preferable for extreme rainfall frequency analysis, the 2010-2024 period was considered acceptable for the objective of this study because it provides a continuous and homogeneous dataset within the available official records, and because the proposed framework is intended for data-scarce regions where long-term sub-daily rainfall series are not available. Nevertheless, we now explicitly acknowledge this record length as a limitation, particularly for estimating very rare return periods such as 1000 years.

The introduction was modified as follows:

Original:Accordingly, this study proposes and validates a method to reconstruct IDF curves exclusively from daily rainfall data. The approach combines temporal disaggregation with Gumbel distribution fitting, requiring only daily maxima and standard statistical tools. It was applied to a 35-year dataset from Habana del Este, a coastal municipality in Cuba, and is intended for broader use in other data-scarce regions where sub-daily observations are lacking. The proposed methodology emphasizes accessibility, simplicity, and replicability in contexts where resource constraints make traditional pluviograph-based analyses impractical.

Modification:Accordingly, this study proposes and validates a method to reconstruct IDF curves from daily rainfall data. The approach combines compatibility screening, temporal disaggregation, Gumbel frequency analysis, Sherman model fitting, and weighted regional unification. It was applied to a 15-year dataset of daily maximum rainfall records from Habana del Este, Cuba, covering the period 2010–2024.

The methodology section was improved as follows:

Original: Daily maximum rainfall data for the period 2010–2024 were obtained from the Sistema de Gestión Integral del Agua (SGIA), Cuba’s official hydrometeorological platform. Initially, ten pluviometric stations located within or near Habana del Este were considered. A preliminary quality check eliminated two stations with insufficient temporal coverage or unreliable records. The remaining eight stations were subjected to homogeneity analysis using the coefficient of variation (CV), comparing the relative dispersion of each series against the group mean. Seven stations met the ±15% homogeneity criterion; the eighth, though initially excluded due to anomalous dispersion, was later reconstructed.

Modification: Daily maximum rainfall data for the period 2010–2024 were obtained from the Sistema de Gestión Integral del Agua (SGIA), Cuba’s official hydrometeorological platform. No extension of the dataset prior to 2010 was performed. Initially, ten pluviometric stations located within or near Habana del Este were considered. A preliminary quality check eliminated two stations with insufficient temporal coverage or unreliable records. The remaining eight stations were subjected to homogeneity analysis using the coefficient of variation (CV), comparing the relative dispersion of each series against the group mean. Seven stations met the ±15% homogeneity criterion; the eighth station, which initially showed anomalous dispersion due to incomplete annual maximum values within the 2010–2024 period, was reconstructed using the mean ratio method and subsequently reevaluated for homogeneity.

The following sentence was added to the methodology as well:

Therefore, the final frequency analysis was based exclusively on annual maximum rainfall series within the 2010–2024 period.

3. Justification for parameter k= 0.45. In line 136, the value of k= 0.45 was used for exponential disaggregation. While this choice may be empirically reasonable, the manuscript would benefit from a brief justification or reference for adopting this specific value. Clarify whether k= 0.45 was derived from calibration with local pluviograph data, from literature specific to tropical climates, or adopted as a standard assumption. This will improve transparency and reproducibility.

We thank the reviewer for this helpful comment. We agree that selecting the disaggregation parameter k = 0.45 required clearer justification. In the revised manuscript, we clarified that k = 0.45 was neither derived from a full local calibration against a complete pluviograph archive nor treated as a universal constant. Instead, it was adopted as an empirical disaggregation exponent suitable for humid tropical rainfall regimes, particularly those influenced by convective and convective stratiform events.

We have revised the Theory and Materials and Methods sections to clarify that this parameter was selected based on hydrological guidance and prior regional applications in tropical rainfall disaggregation. We also clarified that its use was evaluated through the internal consistency of the resulting IDF curves, including smoothness, monotonic decay with increasing duration, and agreement between the disaggregated and unified IDF estimates.

The theory section was improved and modified as follows:

Original: Here, k controls the rate at which rainfall accumulates during a storm. Based on empirical calibration, k=0.45 was adopted in this work. This function allows partitioning daily rainfall totals h24 into shorter durations:

Modification: Here, k controls the rate at which the daily rainfall depth is redistributed into shorter durations. In this study, k = 0.45 was adopted as an empirical disaggregation exponent suitable for humid tropical rainfall regimes, particularly those influenced by convective and convective stratiform storm structures. This value was not treated as a universal constant or as the result of a full local pluviograph calibration. Rather, it was selected based on hydrological guidance and previous regional applications for tropical rainfall disaggregation, and its use was subsequently evaluated by assessing the internal consistency of the resulting IDF curves. This function allows partitioning daily rainfall totals h24 into shorter durations:

The Methodology section was modified as well:

Original:Stations found incompatible with the pluviograph were processed separately using a temporal disaggregation procedure. The adopted model, based on an exponential decay function calibrated with k=0.45, redistributed each 24-hour precipitation total into shorter durations, preserving volume and reflecting typical storm structures in tropical climates.

Modification: Stations found incompatible with the pluviograph were processed separately using a temporal disaggregation procedure. The adopted model used an empirical exponent of k = 0.45 to redistribute each 24-hour precipitation total into shorter durations while preserving the daily rainfall volume. This value was selected because it is consistent with humid tropical rainfall regimes dominated by convective and convective stratiform events, as reported in regional hydrological guidance and applied disaggregation procedures. The parameter was not derived from a full calibration against local pluviograph records; therefore, its use was assessed by examining the smoothness, monotonic behavior, and internal agreement of the resulting IDF curves.

4. Discussion of limitations and implications. In line 282, the authors acknowledge the assumption of a uniform intra-day rainfall distribution as a simplification. This is an important limitation, but its quantitative or conceptual impact on results is not sufficiently discussed. Please elaborate on how this assumption may influence the derived IDF curves, for example, whether it could lead to underestimation of peak intensities at shorter durations or affect design rainfall estimates. Additionally, suggest potential ways to address this limitation in future work.

We expanded the limitations paragraph to clarify that the uniform intra-day rainfall distribution assumption may smooth intense convective rainfall peaks and lead to underestimation of short-duration intensities, particularly for 10, 20, 30, and 60 min events. We also clarified that this may affect design rainfall estimates for small urban drainage structures, culverts, and local stormwater systems. Finally, we added specific future work recommendations, including calibration with longer local pluviograph records, radar rainfall estimates, satellite precipitation products, and stochastic storm profiles. The discussion was improved as follows:

Original: Despite the success of the approac

Attachment Submitted filename: ResponseReviewersComments_21052026.docx

# Editor report: Decision Letter 1

**DOI:** 10.1371/journal.pone.0351841.r003

**Authors:** Nitin Bassi, Nitin Bassi

2 Jun 2026

Reconstructing IDF Curves from Daily Rainfall Records in Data-Scarce Regions: A Statistical Method Based on Temporal Disaggregation and Gumbel Modeling

PONE-D-25-42783R1

Dear Dr. Molino,

We’re pleased to inform you that your manuscript has been judged scientifically suitable for publication and will be formally accepted for publication once it meets all outstanding technical requirements.

Within one week, you’ll receive an e-mail detailing the required amendments. When these have been addressed, you’ll receive a formal acceptance letter and your manuscript will be scheduled for publication.

An invoice will be generated when your article is formally accepted. Please note, if your institution has a publishing partnership with PLOS and your article meets the relevant criteria, all or part of your publication costs will be covered. Please make sure your user information is up-to-date by logging into Editorial Manager at Editorial Manager® and clicking the ‘Update My Information' link at the top of the page. For questions related to billing, please contact billing support.

If your institution or institutions have a press office, please notify them about your upcoming paper to help maximize its impact. If they’ll be preparing press materials, please inform our press team as soon as possible -- no later than 48 hours after receiving the formal acceptance. Your manuscript will remain under strict press embargo until 2 pm Eastern Time on the date of publication. For more information, please contact onepress@plos.org.

Kind regards,

Nitin Bassi

Academic Editor

PLOS One

Additional Editor Comments (optional):

Reviewers' comments:

# Editor report: Acceptance letter

**DOI:** 10.1371/journal.pone.0351841.r004

**Authors:** Nitin Bassi, Nitin Bassi

PONE-D-25-42783R1

PLOS One

Dear Dr. Molino,

I'm pleased to inform you that your manuscript has been deemed suitable for publication in PLOS One. Congratulations! Your manuscript is now being handed over to our production team.

At this stage, our production department will prepare your paper for publication. This includes ensuring the following:

* All references, tables, and figures are properly cited

* All relevant supporting information is included in the manuscript submission,

* There are no issues that prevent the paper from being properly typeset

You will receive further instructions from the production team, including instructions on how to review your proof when it is ready. Please keep in mind that we are working through a large volume of accepted articles, so please give us a few days to review your paper and let you know the next and final steps.

Lastly, if your institution or institutions have a press office, please let them know about your upcoming paper now to help maximize its impact. If they'll be preparing press materials, please inform our press team within the next 48 hours. Your manuscript will remain under strict press embargo until 2 pm Eastern Time on the date of publication. For more information, please contact onepress@plos.org.

You will receive an invoice from PLOS for your publication fee after your manuscript has reached the completed accept phase. If you receive an email requesting payment before acceptance or for any other service, this may be a phishing scheme. Learn how to identify phishing emails and protect your accounts at https://explore.plos.org/phishing.

If we can help with anything else, please email us at customercare@plos.org.

Thank you for submitting your work to PLOS ONE and supporting open access.

Kind regards,

PLOS ONE Editorial Office Staff

on behalf of

Mr Nitin Bassi

Academic Editor

PLOS One
